# Supplementary material for: Splice-Junction-Based Mapping of Alternative Isoforms in the Human Proteome
Source: Cell Rep. Author manuscript; Available in PMC 2020 Jan 15. (PMC6961840; doi:10.1016/j.celrep.2019.11.026)
Supplement: 2 [file NIHMS1546469-supplement-2.zip › DF1/PXD009737/TestisTrypsin/TestisTrypsin_53_UVSSA_CSGAATSLLPGSLSLCS.pdf]

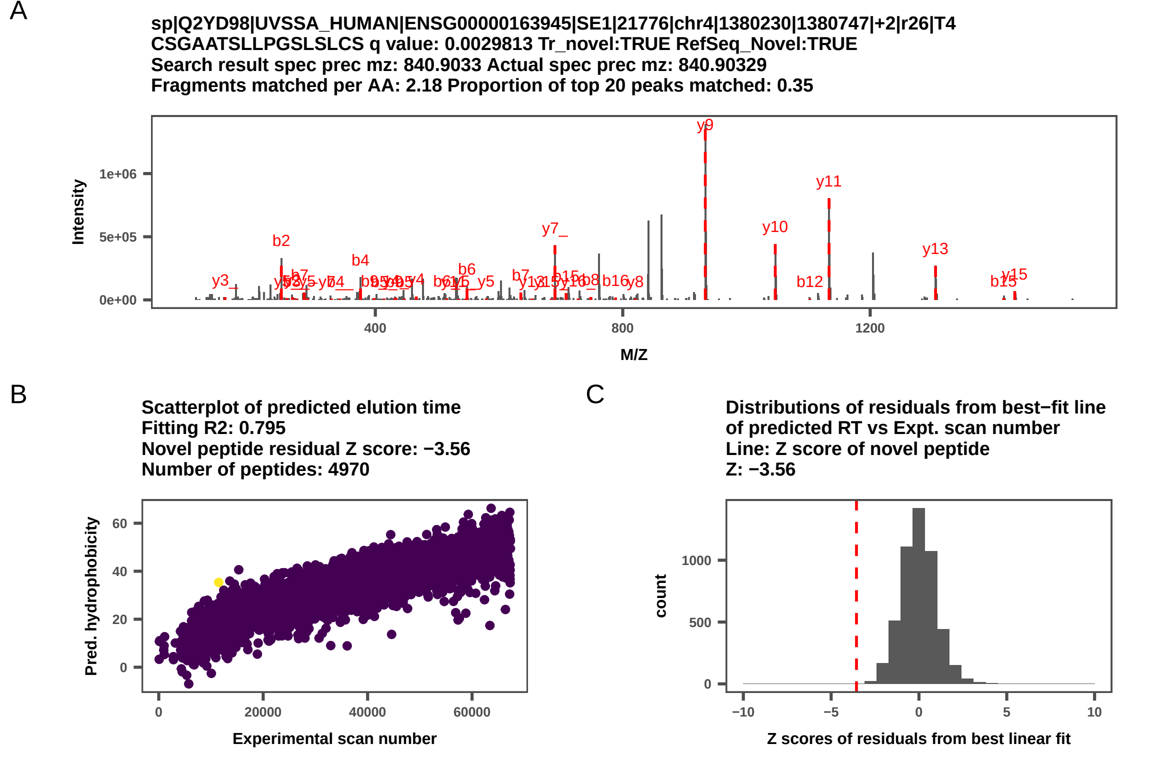
A
sp|Q2YD98|UVSSA_HUMAN|ENSG00000163945|SE1|21776|chr4|1380230|1380747|+2|r26|T4
CSGAATSLLPGSLSLCS q value: 0.0029813 Tr_novel:TRUE RefSeq_Novel:TRUE
Search result spec prec mz: 840.9033 Actual spec prec mz: 840.90329
Fragments matched per AA: 2.18 Proportion of top 20 peaks matched: 0.35
Intensity
1e+06
5e+05
0e+00
y9
y11
y10
y7_
y13
b2
b4
b6
b12
y3_
y8
y15
400
800
1200
M/Z
B
Scatterplot of predicted elution time
Fitting R2: 0.795
Novel peptide residual Z score: −3.56
Number of peptides: 4970
Pred. hydrophobicity
60
40
20
0
0
20000
40000
60000
Experimental scan number
C
Distributions of residuals from best−fit line
of predicted RT vs Expt. scan number
Line: Z score of novel peptide
Z: −3.56
count
1000
500
0
−10
−5
0
5
10
Z scores of residuals from best linear fit
